# Efficacy of Acceptance and Commitment Therapy in Daily Life (ACT-DL) in early psychosis: study protocol for a multi-centre randomized controlled trial

**DOI:** 10.1186/s13063-019-3912-4

**Published:** 2019-12-26

**Authors:** Ulrich Reininghaus, Annelie Klippel, Henrietta Steinhart, Thomas Vaessen, Martine van Nierop, Wolfgang Viechtbauer, Tim Batink, Zuzana Kasanova, Evelyne van Aubel, Ruud van Winkel, Machteld Marcelis, Therese van Amelsvoort, Mark van der Gaag, Lieuwe de Haan, Inez Myin-Germeys

**Affiliations:** 1 Department of Public Mental Health, Central Institute of Mental Health, Medical Faculty Mannheim, University of Heidelberg, Mannheim, Germany; 2 Department of Psychiatry and Psychology, School for Mental Health and Neuroscience, Maastricht University, Maastricht, The Netherlands; 3 ESRC Centre for Society and Mental Health and Centre for Epidemiology and Public Health, Health Service and Population Research Department, Institute of Psychiatry, Psychology and Neuroscience, King’s College London, London, UK; 4 Department of Neurosciences, Psychiatry Research Group, Center for Contextual Psychiatry, KU Leuven, Leuven, Belgium; 5 Universitair Psychiatrisch Centrum KU Leuven, Kortenberg, Belgium; 6 Department of Clinical Psychology, VU Amsterdam, Amsterdam, The Netherlands; 7 Department of Psychiatry, University of Amstderdam, Amsterdam, The Netherlands

## Abstract

**Background:**

Psychotic experiences, social functioning and general psychopathology are important targets for early intervention in individuals with Ultra-High-Risk state (UHR) and a first-episode psychosis (FEP). Acceptance and Commitment Therapy (ACT) is a promising, next-generation Cognitive Behavioural Therapy (CBT) that aims to modify these targets, but evidence on sustainable change and its underlying mechanisms in individuals’ daily lives remains limited. The aim of the INTERACT study is to investigate the efficacy of a novel ecological momentary intervention, Acceptance and Commitment Therapy in Daily Life (ACT-DL) in a multi-centre randomised controlled trial of individuals with UHR or FEP.

**Methods/design:**

In a multi-centre randomised controlled trial, individuals aged 16–65 years with UHR or FEP will be randomly allocated to ACT-DL in addition to treatment as usual (TAU) as the experimental condition or a control condition of TAU only, which will include – for the entire study period – access to routine mental health care and, where applicable, CBT for psychosis (CBTp). Outcomes will be assessed at baseline (i.e. before randomisation), post-intervention (i.e. after the 8-week intervention period), and 6-month and 12-month follow-ups (i.e. 6 and 12 months after completing the intervention period) by blinded assessors. The primary outcome will be distress associated with psychotic experiences, while secondary outcomes will include (momentary) psychotic experiences, social functioning and psychopathology. Process measures to assess putative mechanisms of change will include psychological flexibility, stress sensitivity and reward experiences. In addition, acceptability, treatment adherence and treatment fidelity of ACT-DL will be assessed.

**Discussion:**

The current study is the first to test the efficacy of ACT-DL in individuals with UHR and FEP. If this trial demonstrates the efficacy of ACT-DL, it has the potential to significantly advance the treatment of people with UHR and FEP and, more generally, provides initial support for implementing mHealth interventions in mental health services.

**Trial registration:**

Netherlands Trial Register, ID: NTR4252. Registered on 26 September 2013.

## Background

The state of Ultra-High-Risk (UHR) (also known as an At-Risk Mental State (ARMS) or High-Risk (HR) state) [1, 2] is associated with an increased risk of developing a first-episode psychosis (FEP), with meta-analytic evidence suggesting that conversion to FEP is most likely to occur within 2 years (risk estimate, 29%; 95% CI, 23–36) and to plateau from the third year after presentation to mental health services (risk estimate, 36% after 3 years, approximately 35% after 10 years) [3, 4]. This has been taken to suggest that the UHR state is temporally and phenomenologically continuous with FEP [5], together reflecting the early stages of psychotic disorder [1, 2, 5–7]. Further, social functioning of UHR individuals, who do neither convert to psychosis nor remit, has been reported to be lower than in healthy controls and remarkably similar to those who convert to psychosis [8]. It has been argued that providing an avenue for help in individuals with UHR is important to reduce distress associated with psychotic experiences and impaired functioning to prevent deterioration and persistence prior to the onset of full-blown psychotic symptoms [9]. While sustained periods of remission occur after the first onset of a psychotic disorder [10], persisting psychotic symptoms are associated with significant levels of distress [11, 12] and poor long-term functioning and social outcomes have been reported for the majority of FEP individuals [10, 13], who face a marked mortality gap compared with the general population [14].

A number of psychological mechanisms have been proposed by current aetiological models that may contribute across different phenomenological and temporal stages to the development of psychosis [15–20]. One mechanism that has been repeatedly suggested to play an important role is behavioural sensitisation, which has been posited to amplify the stress response in individuals with increased genetic and/or socio-environmental risk, such that they experience a greater response to even minor stressors and daily hassles, which, in turn, contributes to pushing them along a pathway to psychosis over time [21]. At the behavioural level, the most commonly used marker of this underlying process of behavioural sensitisation is stress sensitivity, characterised by stronger negative emotional reactions to minor stressors in daily life [22, 23]. Previous research suggests that emotional reactivity to minor stressful events, activities, and social situations is increased in individuals with UHR [23, 24] and FEP [23]. At the same time, it has been shown that deficits in reward experience are linked to motivational impairments in psychosis [25, 26].

Developing and evaluating interventions that directly modify these putative mechanisms in daily life to reduce the intensity of psychotic experiences at an early stage is a promising strategy for preventing transition to, and improving outcomes of, psychosis [23, 27–29]. Building on recent advances in the field of mobile Health (mHealth) interventions [30], we have recently proposed an ecological interventionist causal model approach for targeting psychological mechanisms in daily life [29]. This approach draws on ecological momentary interventions (EMIs) (as proposed by our own group [28, 29] and others [31]), which deliver real-time psychological interventions in daily life, thereby, enabling individuals to access interventions that are tailored to what a person needs in a given moment and context, with the goal of producing changes in mechanisms that lead to sustainable change in intended outcomes under real-world conditions [29].

While the initial evidence suggests that psychological interventions such as Cognitive Behavioural Therapy (CBT) may be efficacious in reducing transition rates in individuals with UHR, there is still only a small number of methodologically robust studies to investigate this issue, and evidence on sustainable change in relation to distress associated with symptoms, social functioning as well as the above-mentioned psychological mechanisms remains very limited [32–34]. Recently, there has been increasing interest in Acceptance and Commitment Therapy (ACT), which is a next-generation CBT targeting the relationship of individuals with their feelings and thoughts rather than their content, with the overarching goal of enhancing individuals’ psychological flexibility [35, 36]. ACT aims to train individuals in core psychological processes of acceptance (e.g. of unpleasant, stressful feelings and thoughts), non-judgemental contact with the present moment, values, committed action, self as context, and cognitive defusion [35, 37–39]. While ACT components targeting acceptance are likely to be effective in attenuating stress sensitivity, ACT components targeting commitment (values, committed action) are likely to enhance reward-related motivated action. There is good evidence on the feasibility and acceptability of ACT in people with psychosis [40, 41]. Initial evidence further suggests that ACT may reduce hospital re-admission rates, psychotic and affective symptoms, social impairment, and distress associated with hallucinations in this population [42–45]. While some studies have reported an effect of ACT on hypothesised mechanisms (such as experiential avoidance or belief flexibility about symptoms) [39, 40, 44], a recent RCT in people with persisting psychotic symptoms did not find an effect on targeted mechanisms, calling for improved investigation of psychological processes underlying change in distress and other outcomes [45]. Further, our understanding of whether, and if so how, therapeutic effects translate into individuals’ daily lives remains very limited.

Delivering ACT and evaluating its effects on putative mechanisms in daily life based on principles of EMIs is, therefore, both timely and eminently important. Acceptance and Commitment Therapy in Daily Life (ACT-DL) has recently been developed for enhancing the therapeutic effects of ACT under real-world conditions [28–30, 46]. ACT may be particularly amenable to be implemented as an EMI, as it emphasises the context in which a behavior occurs as well as the function of this behavior in a given context [46]. In a recently completed pilot study to evaluate the acceptability and clinical feasibility of ACT-DL in a heterogeneous clinical sample of patients with mental disorder, very good completion rates, use of exercises, and positive user experience were found [47], but there is no robust, trial-based evidence on its effects in the early stages of psychosis.

Against this background, the aim of the current study is to investigate the efficacy of ACT-DL in a multi-centre randomised controlled trial of patients with UHR or FEP (INTERACT). The manualised ACT-DL intervention will be administered to UHR and FEP patients in addition to treatment as usual (TAU) (experimental condition) and compared to a control condition of TAU only, which will be standard mental health care including Cognitive Behavioural Therapy for psychosis (CBTp) where applicable. Specifically, the study aims to:

1. Test the efficacy of ACT-DL on reducing distress associated with psychotic experiences at post-intervention, 6-month and 12-month follow-ups (primary outcome)
2. Test the efficacy of ACT-DL on reducing (momentary) psychotic experiences, psychopathology, and improving social functioning (secondary outcomes), as well as on reducing stress sensitivity and enhancing reward experience and psychological flexibility (process measures to assess mechanisms of change) at post-intervention, 6-month, and 12-month follow-ups
3. Examine, consistent with established credibility criteria [48], the effects of ACT-DL in UHR compared with FEP individuals in a priori planned subgroup analyses
4. Assess the acceptability, treatment adherence and treatment fidelity of ACT-DL in UHR and FEP patients

## Methods/design

### Study design

**Fig. 1:**
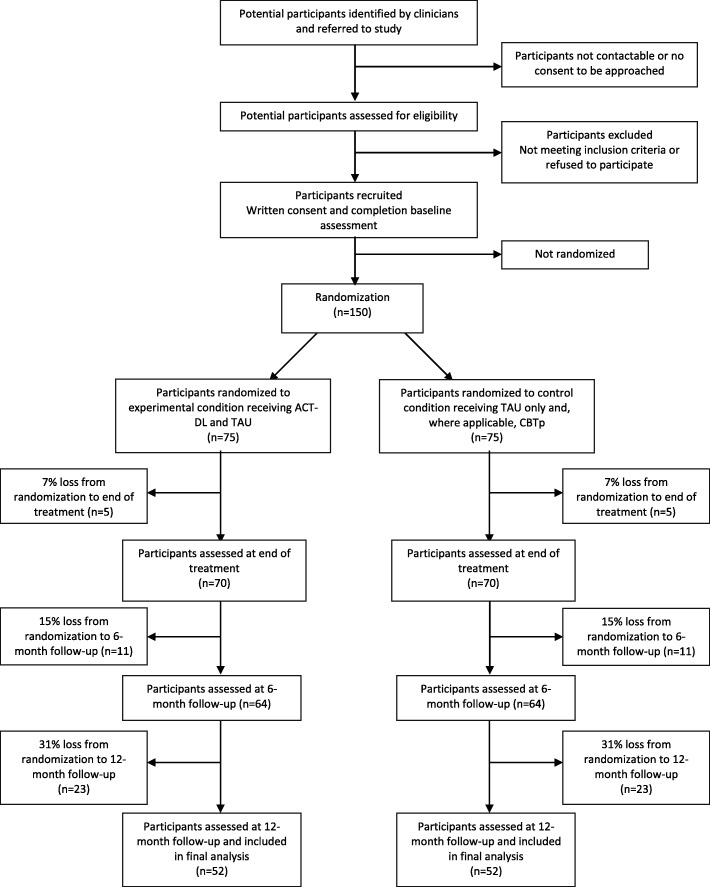
Anticipated study flowchart

**Fig. 2:**
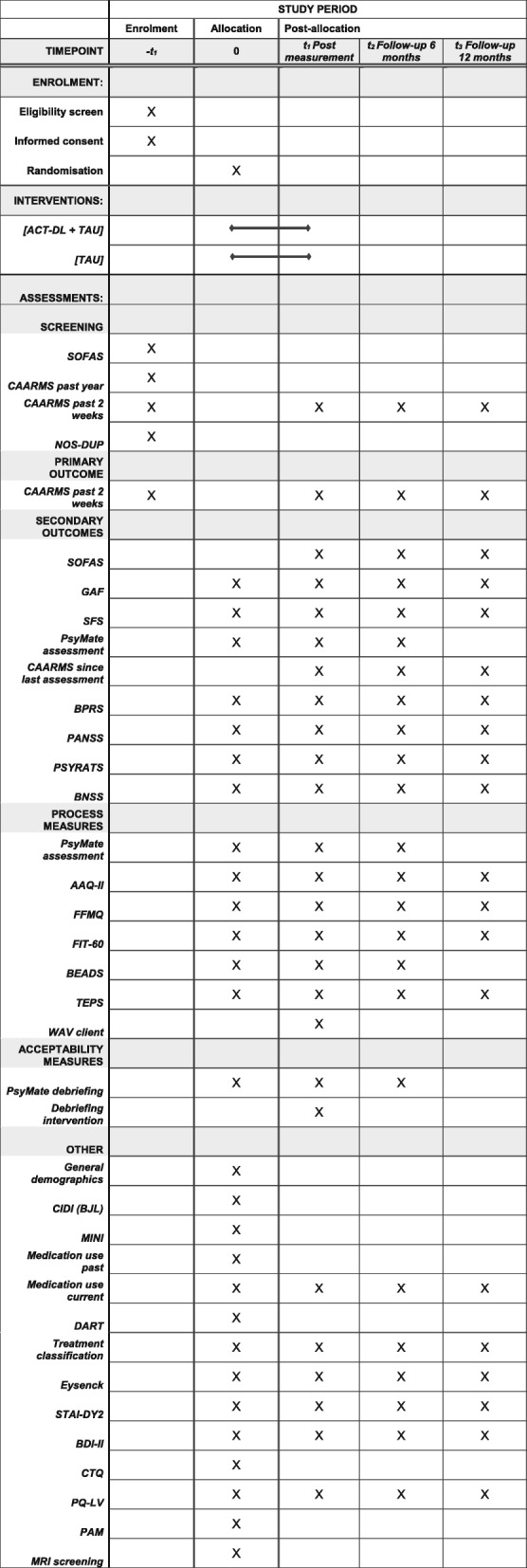
Standard Protocol Items: Recommendations for Interventional Trials (SPIRIT) Figure

In a multi-centre randomised controlled trial, individuals aged 16–65 years with UHR or FEP will be randomly allocated to ACT-DL in addition to TAU as the experimental condition or a control condition of TAU only, which will include routine mental health care and, where applicable, CBTp. Participants will be recruited from mental health services in the Netherlands and Flanders, Belgium. Outcomes will be assessed at baseline (i.e. before randomisation), post-intervention (i.e. after the 8-week intervention period), and 6-month and 12-month follow-ups (i.e. 6 and 12 months after completing the intervention period) by blinded assessors (see Figs. 1 and 2, and Additional file 1 in Supplementary information). Randomisation will be conducted by an independent researcher through a computer-generated sequence. All outcomes will be assessed and all statistical analyses will be conducted blind to treatment allocation.

### Participants

The study will aim to recruit 150 participants with UHR or FEP from secondary mental health services at clinical sites of five centres: (1) Amsterdam (Academic Medical Centre, Arkin Basis GGZ), (2) The Hague (Parnassia/PsyQ), (3) Maastricht/Eindhoven (Mondriaan, Virenze, GGZE) (all in the Netherlands), (4) Flemish-Brabant (Leuven (UPC KU Leuven), Antwerp (VDIP), Diest (Sint-Annendael), Mortsel (PCM)), and (5) East/West Flanders (Brugge (OLV), Melle (Karus), Sint Niklaas (VDIP)) (all in Belgium). Individuals receiving care from these secondary mental health services will be approached by a clinician of these services, who will provide initial information about the study. Individuals, who are interested to take part in the study, will be asked for their consent to be approached by a member of the research team to learn more about the study. If the potential participant agrees, they will be fully informed about the study in a face-to-face session or on the phone by a researcher and, after 1 week, asked for written informed consent. Full eligibility assessment will be conducted by the researcher once participants have provided written informed consent, which can be withdrawn at any time without any negative consequences for participants. Participants will be rewarded financially for complete participation, and travel expenses will be fully reimbursed.

#### Inclusion criteria

The inclusion criteria are as follows: (1) aged 16–65 years; (2) an UHR (without prior use of antipsychotic medication) or FEP (onset within last 3 years) as assessed by the Comprehensive Assessment of At Risk Mental State (CAARMS) [1] and Nottingham Onset Schedule (NOS) [49]; (3) sufficient command of the Dutch language to follow instructions for assessment of outcomes and receiving the intervention; and (4) ability to provide written informed consent.

#### Exclusion criteria

The exclusion criteria are as follows: (1) a primary diagnosis of alcohol/substance abuse and dependence, assessed with the Mini-International Neuropsychiatric Interview (MINI) [50]; and (2) severe endocrine, cardiovascular or brain disease.

### Interventions

#### Control condition: treatment as usual (TAU)

Participants allocated to the treatment as usual (TAU) control condition will continue to receive all the treatment they received prior to the start of the study. This will include good standard care delivered according to local and national service guidelines and protocols by their general practitioner, psychiatrist and other members of the mental health care team. Standard mental health care will include CBTp at some sites, which will be assessed together with other service contacts using a service-use checklist to monitor variation in the delivery of mental health services and allow for planned subgroup analysis.

#### Experimental condition: ACT-DL + TAU

Participants allocated to the experimental condition will receive ACT-DL with trained clinicians within an 8-week period in addition to TAU, which will consist of all the treatment that they received prior to the start of the study and include all the input from their general practitioner, psychiatrist, and other members of the mental health care team that they would receive if they did not participate in the study, with the exception of manualised CBTp. The intervention can be discontinued by participants at any time upon request without any negative consequences.

The manualised ACT-DL intervention consists of eight ACT training sessions (including one session for psychoeducation) administered face-to-face by a trained clinician (i.e. psychologists having received a 5-day training in ACT-DL and receiving fortnightly supervision sessions for the purposes of the trial), each for around 45–60 min, and an ACT-based EMI, which participants will receive following randomisation to the experimental condition [46], over an 8-week intervention period. The latter will be administered through a smartphone-based app (i.e. the PsyMate™ app) to allow participants to apply the skills that they have been trained into their daily lives [28–30]. The first six face-to-face ACT sessions are based on a modified version of ACT for people with psychosis [35, 43, 44, 51] and aim to enhance participants’ psychological flexibility by training them in six core components: creative hopelessness, acceptance, cognitive defusion, self as context and contact with the present moment, values, and committed action [46, 52]. In the last session, all six components will be integrated and reviewed.

The ACT-based EMI will train participants in applying the ACT techniques and skills from the sessions to their daily life through exercises and metaphors focussing on the six ACT components without involvement of the trained clinician on at least three consecutive days per week following (from session 2) each face-to-face session. On each of these days, participants receive prompts on the app at eight semi-random moments, asking them to complete a brief questionnaire on their current mood, psychotic experiences and activities, with the goal of increasing awareness of their current psychological state. Participants are then offered either an ACT exercise or metaphor training them in the ACT component covered in the face-to-face session. After participants are trained in each ACT component separately, the EMI is extended to cover the full range of components in order to train participants to flexibly adopt ACT skills and techniques depending on the context. In addition, participants are asked to apply skills and techniques in situations when they are most needed (e.g. at times of distress associated with psychotic experiences, during challenging activities or situations). After completion of the intervention period, participants will no longer have access to the app. Please see Steinhart et al. [46] for a more detailed account of the ACT-DL intervention.

### Outcomes

Following written informed consent and full eligibility assessment, all eligible patients will be assessed on all outcomes before randomisation (‘baseline’), after the 8-week intervention period (‘post-intervention’), and after a 6-month and 12-month follow-up periods (‘follow-up’) by blinded assessors (see Fig. 1). Secondary outcomes and process measures using the Experience Sampling Method (ESM) will be assessed at baseline, post-intervention, and 6-month follow-up.

#### Primary outcomes

The primary outcome of the study is distress associated with psychotic experiences measured with the mean distress score of the CAARMS positive symptom subscale (range 0–100) [1]. The CAARMS is a semi-structured interview, which is sensitive to change [33] and shows a high reliability [53].

#### Secondary outcomes

The secondary outcomes of the study are global and social functioning, (momentary) psychotic experiences, and psychopathology. Measures to assesses secondary outcomes will include the Global Assessment of Functioning (GAF) [54] scale, the Social and Occupational Functioning Assessment Scale (SOFAS) [55], and the Social Functioning Scale (SFS) [56] to assess global and social functioning. In addition, the Experience Sampling Method (ESM), a structured, random time-sampling diary technique will be used to measure activities and social contacts ten times per day over a period of six consecutive days using an established ESM data collection protocol on a smartphone-based app (the PsyMate™ app) [22–24, 57–60] in order to assess momentary social functioning [61]. Secondary outcome measures will further include the Brief Psychiatric Rating Scale (BPRS) [62] and the Brief Negative Symptom Scale (BNSS) [63] to cover the full range of psychotic experiences and psychopathology as well as the CAARMS and Positive and Negative Syndrome Scale (PANSS) [64] for a-priori planned subgroup analyses in UHR and FEP participants. Also, psychopathology will be assessed in daily life with ESM (including momentary psychotic experiences and momentary negative affect).

#### Process measures

Process measures to assess putative mechanisms of change will include ESM measures of minor stressors, negative affect, pleasantness of events, and positive affect to assess stress sensitivity (operationalised as an increase in negative affect in response to minor stressors) and reward experience (operationalised as an increase in positive affect in response to pleasant events) at baseline, post-intervention, and 6-month (and – for non-ESM measures – 12-month) follow-up [22–24, 57–60]. Psychological flexibility operationalised as the six core ACT competences (see above) will be measured using the Acceptance and Action Questionnaire [65, 66], the Five Facet Mindfulness Questionnaire [67], the Flexibility Index Test [68], and the ESM. Cognitive flexibility will be measured using the PSYRATS to assess belief flexibility [69], the beads task to assess reasoning bias [70], and experimental Experience Sampling Methodology (eESM) task for measuring liberal acceptance bias [71]. In addition, the Temporal Experience of Pleasure Scale (TEPS) [72] will be used to assess anticipatory and consummatory pleasure and, more broadly, reward experience. The therapeutic alliance will be assessed using the Working Alliance Inventory [73, 74] and will involve clinician and patient ratings. In addition, the app-based EMI of ACT-DL will provide detailed process measures of mood, psychotic experiences and activities in the experimental condition.

#### Acceptability, treatment adherence and treatment fidelity

Acceptability of ACT-DL will be assessed at post-intervention through a questionnaire asking participants to evaluate ease of use, accessibility and comprehensiveness of various components of the intervention. The app-based EMI of ACT-DL will further provide detailed data on treatment adherence to ACT-DL (e.g. number of exercises completed per week). Treatment fidelity will be rated based on a random selection of audio tapes of three training sessions recorded by clinicians delivering ACT-DL using an ACT-DL adherence checklist covering all core ACT and EMI components [46].

#### Other measures

Other measures will assess socio-demographic characteristics, alcohol/substance use (Composite International Diagnostic Interview [75], MINI [50]), current and past medication use, and IQ (Dutch Adult Reading Test [76]) as potential confounders that may be associated with primary and secondary outcomes. Service use will be assessed using a therapy classification checklist. Also, personality (Eysenck Personality Questionnaire [77]), trait anxiety (State-Trait Anxiety Inventory [78]), depression (Beck Depression Inventory-II [79]), psychotic experiences (Prodromal Questionnaire Long Version [80]), attachment (Psychosis Attachment Measure [81]), and childhood trauma (Childhood Trauma Questionnaire [82]) will be assessed.

### Sample size

Previous studies suggest that third-wave CBT [40, 83, 84], including ACT [40, 44], may yield reductions in psychotic experiences of moderate to large effect size. Consistent with previous research [44], the power calculation is based on the primary outcome of a reduction in distress associated with psychotic experiences of moderate effect size (i.e. in line with Gaudiano and Herbert [44]) measured with the CAARMS. Power simulation in R indicates that a sample size of *n* = 150 participants (75 experimental, 75 control condition) will be sufficient to test our primary hypothesis of the effect of condition (ACT-DL + TAU vs. TAU) on distress associated with psychotic experiences at all three time points (i.e. post-intervention, 6-month and 12-month follow-ups), which will be tested using an omnibus test of no difference between the two conditions at all three time points against the two-sided alternative hypothesis that there is a difference at one (or more) of the three follow-up time points, while controlling for baseline distress associated with psychotic experiences. Specifically, we expect an attrition rate of 31%, resulting in a loss to follow-up of 23 individuals per condition on average. Hence, we will recruit a total sample of *n* = 150 participants (75 per arm) at baseline to the study, which allows for a 31% attrition rate and leaves *n* = 104 participants to detect a medium effect size of *d* = 0.5 at (at least) one of the post-intervention and follow-up time points, with a power of 0.92 when testing at alpha = 0.05. Power simulation further indicates that a sample of 150 participants, will be sufficient to detect a large effect size (Cohen’s *d* = 0.8) at *p* < 0.05 for the difference in the effect of condition on distress associated with psychotic experiences between FEP and UHR with a power of 0.75 at post-intervention and follow-up in a-priori planned subgroup analysis, while allowing, again, for a 31% attrition rate (notably, given the power calculation expected this attrition rate to be constant at all three time points and not to increase – as is expected – over time (see Fig. 1), power was under-estimated for this secondary analysis). Hence, this sample size will allow us to test the secondary hypothesis whether there is a clinically meaningful difference (of large effect size) between FEP and UHR that would be relevant to be considered in the implementation of ACT-DL in routine care for these patient groups.

### Randomisation and blinding

Participants will be randomised at a 50:50 ratio to the experimental or control condition at the level of the individual participant by an independent researcher through a computer-generated sequence following informed consent, full eligibility assessment, and assessment of all outcome measures. Block randomisation will be carried out in blocks of six participants, with stratification for the five centres (Amsterdam, The Hague, Maastricht/Eindhoven, Flemish-Brabant, East/West Flanders) and two groups of UHR and FEP (expecting a 50:50 ratio of UHR and FEP to be included in the sample). The researchers will be blind to the allocation of participants to the experimental and control group of the study. There will be a contact person for any questions regarding the procedure that is not involved in any testing to allow researchers to be blind towards the allocation of participants when assessing outcomes. Any breaks in blindness will be documented and another researcher will be allocated to complete the next set of assessments where possible.

### Assessment of safety

We will monitor and record any serious adverse events throughout the entire study period. These are any serious untoward incidents that result in death, persistent or significant disability or incapacity, require (extension of) hospitalisation or are life-threatening. Serious adverse events are not expected to occur as a result of the intervention. All serious adverse events will be reported to the accredited Medical Ethics Review Committees (MERC). If there are concerns about unexpectedly high rates of serious adverse events, this will be investigated further in interim analyses and if this yields any safety or ethical concerns the Trial Management Committee will terminate the trial prematurely.

### Statistical analysis

The investigators will access the final trial dataset to test the primary hypothesis of a reduction in distress associated with psychotic experiences measured with the CAARMS using a linear regression model with distress at all three time points (i.e. post-intervention, 6-month and 12-month follow-ups) as the dependent variable and distress at baseline, condition (ACT-DL + TAU vs. TAU), time (as a three-level factor), centre (as a five-level factor), the baseline × time interaction, and the condition × time interaction as independent variables, according to the intention-to-treat principle. Within-subject clustering of repeated measures will be taken into account by allowing residuals within subjects to be correlated with a completely unstructured variance-covariance matrix.

The model will be fitted using restricted maximum likelihood estimation using Stata 15 [85]. This will allow for the use of all available data under the assumption that data is missing at random and if all variables associated with missing values are included in the model [86, 87]. Hence, bias due to attrition over time, due to difference between groups, or as a function of baseline distress is already mitigated by the model. Potential bias due to missing outcomes will be assessed in descriptive analyses of baseline characteristics stratified by missing data for the primary outcome and condition as follows: (a) experimental condition with no missing primary outcome at post-intervention time point, (b) experimental condition with missing primary outcome at post-intervention time point, (c) control condition with no missing primary outcome at post-intervention time point, and (d) control condition with missing primary outcome at post-intervention time point [88, 89].

The main effect of condition will be tested via an omnibus test of no difference between the two conditions at all three time points (Wald-type test with df = 3 and alpha = .05). Should the omnibus test be statistically significant, then the three time-specific contrasts will be examined to determine at which time points significant differences are present (each tested at alpha = .05). Secondary hypotheses and analyses of process measures to assess putative mechanisms of change will be tested following the same steps. Given that block randomisation will be carried out in blocks of six participants, with stratification for centre and group, all analyses will include centre and group as covariate, even though there is little reason to expect noteworthy clustering of outcomes by centre.

In addition, multilevel mediation analysis will be used to test indirect effects of condition on primary outcomes (distress associated with psychotic experiences) and secondary outcomes (psychotic experiences, psychopathology, social functioning) via pathways through putative mechanisms of change (psychological flexibility, stress sensitivity, reward experience). Multilevel mediation models will be fitted in MPlus, Version 7 [90], to control for within-subject clustering of multiple time points [91, 92], using the MLR estimator, which allows for the use of all available data under the assumption that data is missing at random (if all variables associated with missing values are included in the model). In a two-level model, multiple time points (level 1) will be treated as nested within subjects (level 2). The total effect of condition (level 2) on primary/secondary outcomes (level 1) will be apportioned into direct and indirect (or, synonymously, mediating) effects through putative mechanisms of change (level 1) using the product of coefficients strategy. This strategy quantifies the point estimate of the indirect effect as the product of the coefficient of independent variable on mediator variable (path a) and the coefficient of mediator variable on dependent variable (path b). We will use statistical software by Selig and Preacher [93] for computing Monte Carlo confidence intervals and assessing the statistical significance of indirect effects, given their advantages over rival methods in the context of multilevel mediation models [93, 94].

For analysis involving ESM variables, multiple ESM observations (level 1) will be treated as nested within time points (baseline, post-intervention, 6-month follow-ups) (level 2) and time points as nested within subjects (level 3). Consistent with established credibility criteria [48], we will further test the effects of ACT-DL in UHR compared with FEP individuals in a-priori planned subgroup analyses. For subgroup analyses comparing UHR and FEP, data on the group variable (UHR, FEP) will be measured prior to randomisation (to address the criterion that the subgroup characteristic is measured at baseline) to investigate whether there is a difference in the reduction in distress associated with psychotic experiences measured with the CAARMS of large effect size between UHR and FEP participants (to address the criterion that the expected difference/effect size is specified a priori) given that only a large effect size would be relevant for implementation of ACT-DL in routine care. It will further be examined whether this effect is (a) consistent across (primary and secondary) outcomes and (b) supported by indirect evidence on putative mechanisms of change (psychological flexibility, stress sensitivity, reward experience). In more exploratory sensitivity analyses, we will compare ACT-DL + TAU, CBTp + TAU and TAU only to investigate whether the reduction in distress associated with psychotic experiences measured with the CAARMS will be greater for CBTp + TAU than TAU only as well as for ACT-DL than CBTp.

### Research governance

Maastricht University is the study sponsor. The trial has received a favourable ethical opinion from the MERC at Maastricht University Medical Centre (MUMC), the Netherlands (reference: NL46439.068.13) and the University Clinic Leuven, Belgium (reference: B322201629214). Any amendments to the study protocol will be submitted to the MERC for approval and then communicated to the sponsor, funder, and centres. The protocol will also be updated in the clinical trial registry. Any deviations from the study protocol will be fully documented using a breach report form. The principal investigator (PI) will have overall responsibility for the trial and will be supported by a dedicated research coordinator in the day-to-day management of the trial. The PI will lead the trial coordinating centre and, together with the research coordinator, liaise closely with the site coordinators on recruitment and consent procedures. The Trial Management Committee will meet on a monthly basis and consist of all investigators, the research coordinator and site coordinators. It will be chaired by the PI and manage the day-to-day running of the study, audit the trial conduct, and oversee preparation of reports to the MERC. The PI will permit audits, monitoring and MERC review. Data monitoring and auditing of RCTs approved by MERC at MUMC is conducted by the Clinical Trial Center Maastricht, which is independent from the study sponsor (i.e. Maastricht University). The handling of the data complies with the Dutch and Belgian Personal Data Protection Act. If a participant decides to withdraw their consent, all data from that participant will be destroyed. This trial does not involve collecting biological specimens for storage. Data will be handled confidentially and coded using a number indicating the order of entry. All materials will be securely stored in line with the European General Data Protection Regulation (GDPR), with personnel data stored separately from the number-coded data. We will closely liaise with service user researchers on dissemination activities throughout the trial.

## Discussion

Psychotic experiences (in particular, distress associated with these), social functioning, and psychopathology are important targets for early intervention in individuals with UHR and FEP [9–13]. ACT is a promising, next-generation CBT for reducing distress associated with psychotic experiences, social functioning and psychopathology, but evidence on sustainable change in individuals’ daily lives, and investigations of the putative mechanisms underlying such change in distress and other outcomes, remains limited [32, 33]. Acceptance and Commitment Therapy in Daily Life (ACT-DL) has recently been developed for enhancing the therapeutic effects of ACT and achieving sustainable change in individuals’ daily life [28–30, 46]. The current study is the first to test the efficacy of ACT-DL in a multi-centre randomised controlled trial of patients with UHR or FEP and includes a detailed investigation of process measures of putative mechanisms of change, acceptability, treatment adherence and treatment fidelity. If this trial demonstrates the efficacy of ACT-DL, this has the potential to significantly advance the treatment of people with UHR and FEP and, more generally, provides initial support for implementing mHealth interventions in early intervention services. Findings on putative mechanisms of change will, at the same time, allow us to assess important criteria for establishing causality under real-world conditions [29]. Potential implementation of ACT-DL in early intervention services will be informed by detailed data on its acceptability, treatment adherence and treatment fidelity.

## Trial status

This trial is ongoing. The trial started recruitment in November 2016 and recruitment and outcome assessment will continue until June 2020. The results will be published in peer-reviewed journals in 2020.

## Supplementary information

**Additional file 1.** Standard Protocol Items: Recommendations for Interventional Trials (SPIRIT) 2013 Checklist.

## Data Availability

The datasets generated during and/or analysed during the current study are not publicly available but are available from the corresponding author on reasonable request. The full protocol and statistical code will also be made available upon reasonable request.
