# Correction to: Efficacy of Acceptance and Commitment Therapy in Daily Life (ACT-DL) in early psychosis: study protocol for a multi-centre randomized controlled trial

**DOI:** 10.1186/s13063-021-05513-0

**Published:** 2021-08-16

**Authors:** Ulrich Reininghaus, Annelie Klippel, Henrietta Steinhart, Thomas Vaessen, Martine van Nierop, Wolfgang Viechtbauer, Tim Batink, Zuzana Kasanova, Evelyne van Aubel, Ruud van Winkel, Machteld Marcelis, Therese van Amelsvoort, Mark van der Gaag, Lieuwe de Haan, Inez Myin-Germeys

**Affiliations:** 1 Department of Public Mental Health, Central Institute of Mental Health, Medical Faculty Mannheim, University of Heidelberg, Mannheim, Germany; 2 Department of Psychiatry and Psychology, School for Mental Health and Neuroscience, Maastricht University, Maastricht, The Netherlands; 3 ESRC Centre for Society and Mental Health and Centre for Epidemiology and Public Health, Health Service and Population Research Department, Institute of Psychiatry, Psychology and Neuroscience, King’s College London, London, UK; 4 Department of Neurosciences, Psychiatry Research Group, Center for Contextual Psychiatry, KU Leuven, Leuven, Belgium; 5 Universitair Psychiatrisch Centrum KU Leuven, Kortenberg, Belgium; 6 Department of Clinical Psychology, VU Amsterdam, Amsterdam, The Netherlands; 7 Department of Psychiatry, University of Amstderdam, Amsterdam, The Netherlands

**Correction to: Trials 20, 769 (2019)**

**https://doi.org/10.1186/s13063-019-3912-4**

Following the publication of the original article [1], the authors discovered a typographical error in the method section under the inclusion criteria subsection. The text “[the] inclusion criteria are as follows: (1) aged 16-65 years” should read “[the] inclusion criteria are as follows: (1) aged 15-65 years”. The authors were granted ethical approval for this age range by the MERC at Maastricht University Medical Centre (MUMC), The Netherlands reference: (NL46439.068.13) and the University Clinical Leuven, Belgium (reference: B322201629214). The typographical error therefore has had no implications for the RCT.
